# Use of Causal Diagrams for Nursing Research: a Tool for Application in Epidemiological Studies

**DOI:** 10.17533/udea.iee.v37n1e01

**Published:** 2019-01-06

**Authors:** Wilson Cañón Montañez, Alba Luz Rodríguez Acelas

**Affiliations:** 1 Nurse, Ph.D. Associate Professor, Universidad de Antioquia, Colombia. email: wilson.canon@udea.edu.co Universidad de Antioquia Universidad de Antioquia Colombia wilson.canon@udea.edu.co; 2 Nurse, Ph.D. Associate Professor, Universidad de Antioquia, Colombia. email: aluz.rodriguez@udea.edu.co Universidad de Antioquia Universidad de Antioquia Colombia aluz.rodriguez@udea.edu.co

Many epidemiological studies seek to assess the effect of one or several exposures on one or more outcomes. However, to quantify the causal inference produced, statistical techniques are commonly used that contrast the association among the variables of interest, not precisely of causal effect. (1) In fact, although these measures may not have a causal interpretation, the results are often adjusted for all potential confounding factors.(2^,^^3^) Some contemporary epidemiologists developed new methodological tools for causal inference, like the theory or contra-factual model (4)) and representation of causal effects through the Directed Acyclic Graph (DAG).(5) The DAG, a fusion of the probability theory with trajectory diagrams, is quite useful to visually deduct the statistical associations implied by the causal relations among the study variables.

Learning the rules to visualize causal relations through a DAG can take some time and practice. Once these rules are mastered, they facilitate many tasks, like understanding confusion and selection biases, selecting covariates for statistical adjustment and analysis, understanding direct effects,(6) and analyzing instrumental variables.(7) In this regard, it should be noted that some researchers interested in facilitating the use of causal diagrams and diminish the risk of bias in the epidemiological studies developed the "DAGitty" open software.(8)

It is appropriate to mention that the study of causal mechanisms of health problems constitutes a challenge that, in some scenarios, is often left aside. Although the potential of using DAG is recognized in the hypothesis description of the possible causal networks between the study variables and the presentation of more robust results for the scientific community, I consider, through my personal experience, that these graphs are still used scarcely in research work. This makes it a priority for scientific societies and academic institutions to teach this methodological tool during the formation of researchers undergoing epidemiological studies. Currently, it is possible for some editors and reviewers of scientific indexed journals to ask authors seeking to report results of epidemiological studies to include the DAG in the article. Within this context, a study using data from the National Health and Nutrition Examination Surveys (NHANES) in the United States, and whose objective was to examine the role of serum bilirubin as likely risk factor for hypertension, published in its article the DAG performed in DAGitty to a minimal sufficient adjustment set of variables that permit identifying the true effect, without confusion, of bilirubin in blood pressure.(9) A study from 2018, with data from an epidemiological study in Brazil,(10) reported that metabolic syndrome was associated independently with the global longitudinal strain variation or myocardial deformation index. For example, Figure 1 shows the DAG that represents the conceptual framework, the possible causal relations of the variables and their roles in the association the researchers sought to demonstrate. In this diagram, the exposure variable corresponds to the metabolic syndrome and the outcome is the global longitudinal strain. The other co-variables appearing in the DAG can be classified in several roles, for example: confusion, mediator, proxy confusion, competitive exposure, and collider. To describe the relationships among the variables in a DAG, these can be read as an ancestry tree and kinship terminology is used: child, parent, descendants, and ancestors.(5^,^^8^)

**Figure 1:**
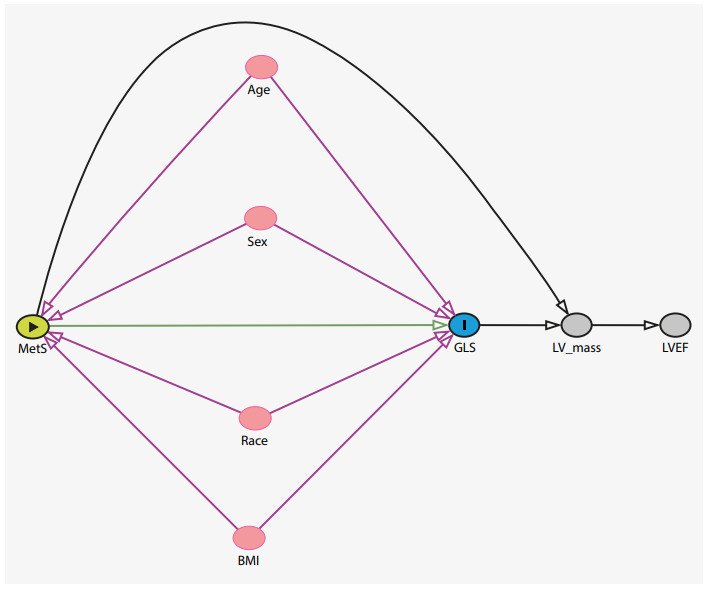
Direct acyclic graph to represent the relationship between metabolic syndrome and global longitudinal strain. Exposure variable: metabolic syndrome (MetS); outcome variable: global longitudinal strain (GLS); confounder variables: age, sex, race, and body mass index (BMI); collider variable: left ventricular mass (LV mass); other variable: left ventricular ejection fraction (LVEF).

Lastly, it is worth highlighting that causal diagrams may also generate useful conclusions, even in situations where it is not possible to identify a sufficient set of variables to control and prevent confusion and selection bias. It is necessary, during the planning phase of epidemiological studies, for researchers to have sufficient knowledge and conceptual framework of all possible variables that can influence the relationship between the exposure and the outcome of interest. This is for the purpose of constructing plausible causal models that permit identifying the variables required to solve the research question and the methodological design that must be used to conduct the study. In addition, using DAGs in communicating and reporting results permits comparing models of causal effects, facilitating identification of possible explanations for the inconsistent results found in the literature. 

Using the DAGs methodology is an opportunity for Nursing in improving knowledge of phenomena and health problems, which will contribute to identifying the necessary elements to be intervened to improve the wellbeing of the population. It is necessary for Nursing graduate programs to adopt these new tools in their study plans to train a new generation of researchers who are at the forefront of methods to analyze causal inference and produce professional progress towards excellence.

## References

[1] Hernán MA, Robins JM (2019). Causal Inference.

[2] Arah OA (2017). Bias Analysis for Uncontrolled Confounding in the Health Sciences. Annu. Rev. Public Health.

[3] VanderWeele TJ, Ding P (2017). Sensitivity Analysis in Observational Research: Introducing the E-Value. Ann. Intern. Med.

[4] Höfler M (2005). Causal inference based on counterfactuals. BMC Med. Res. Methodol.

[5] Cortes TR, Faerstein E, Struchiner CJ (2016). Utilização de diagramas causais em epidemiologia: um exemplo de aplicação em situação de confusão. Cad. Saude Publica.

[6] Aalen OO, Røysland K, Gran JM, Kouyos R, Lange T (2016). Can we believe the DAGs? A comment on the relationship between causal DAGs and mechanisms. Stat. Methods Med. Res.

[7] Langdon RJQ, Wade KH (2017). Application of Mendelian randomization: can we establish causal risk factors for type 2 diabetes in low-to-middle income countries?. Rev. Cuid.

[8] Textor J, van der Zander B, Gilthorpe MS, Liskiewicz M, Ellison GT (2016). Robust causal inference using directed acyclic graphs: the R package 'dagitty'. Int. J. Epidemiol.

[9] Wang L, Bautista LE (2015). Serum bilirubin and the risk of hypertension. Int. J. Epidemiol.

[10] Cañon-Montañez W, Santos ABS, Nunes LA, Pires JCG, Freire CMV, Ribeiro ALP (2018). Central Obesity is the Key Component in the Association of Metabolic Syndrome with Left Ventricular Global Longitudinal Strain Impairment. Rev. Esp. Cardiol.

